# Acceptability and feasibility of magnetic femoral nerve stimulation in older, functionally impaired patients

**DOI:** 10.1186/s13104-018-3493-4

**Published:** 2018-06-15

**Authors:** Louise A. Beveridge, Rosemary J. G. Price, Louise A. Burton, Miles D. Witham, Allan D. Struthers, Deepa Sumukadas

**Affiliations:** 1 Department of Medicine for the Elderly, NHS Tayside, Dundee, Scotland, UK; 2 Division of Molecular and Clinical Medicine, Ninewells Hospital, University of Dundee, Dundee, Scotland, UK; 3 Medicine for the Elderly, Perth Royal Infirmary, Perth, PH1 1NX Scotland, UK

**Keywords:** Older, Magnetic stimulation, Acceptability, Feasibility, Reproducibility

## Abstract

**Objective:**

Magnetic femoral nerve stimulation to test muscle function has been largely unexplored in older people. We assessed acceptability, feasibility, along with reproducibility and correlation with other physical function measures.

**Results:**

Study 1 recruited older people with sarcopenia. Stimulation was performed at baseline and 2 weeks along with six minute walk (6MW), maximum voluntary quadriceps contraction, short physical performance battery and grip strength. Acceptability was measured using visual analog scales. Study 2 used baseline data from a trial of older people. We correlated stimulation results with 6MW, maximal voluntary contraction and muscle mass. Maximum quadriceps twitch tension was measured in both studies, evoked using biphasic magnetic stimulation of the femoral nerve. In study 1 (n = 12), magnetic stimulation was well tolerated with mean discomfort rating of 9% (range 0–40%) on a visual analog scale. Reproducibility was poor (intraclass correlation coefficient 0.06; p = 0.44). Study 2 (n = 64) showed only weak to moderate correlations for maximum quadriceps twitch tension with other measures of physical function (6 minute walk test r = 0.24, p = 0.06; maximal voluntary contraction r = 0.26; p = 0.04). We conclude that magnetic femoral nerve stimulation is acceptable and feasible but poorly reproducible in older, functionally impaired people.

## Introduction

Sarcopenia—the age-related loss of muscle mass and function—is a major health problem leading to increased disability, morbidity and mortality in older people. Determining the effect of interventions for sarcopenia in older people is challenging. Muscle function testing relies heavily on voluntary effort [1–3]. Magnetic nerve stimulation is a relatively unexplored method of objectively assessing muscle function. It offers the advantage of measuring muscle strength and fatigability non-invasively [4, 5]. Unlike exercise and many voluntary strength tests, magnetic nerve stimulation is performed with the individual rested supine and allows measurement of skeletal muscle function while minimising cardiovascular effort. Maximum twitch generated in the quadriceps through magnetic stimulation (TwQ) has been reported to be more reproducible and more sensitive to change than maximum voluntary muscle strength in some populations [6, 7] and has been used successfully in a variety of populations including healthy older people [8]. However no studies have explored magnetic femoral nerve stimulation in older people with impaired physical function.

We therefore examined (a) the acceptability of magnetic femoral nerve stimulation in older, functionally impaired people, (b) the relationship between TwQ and other measures of muscle mass and function, and (c) the reproducibility of TwQ in older, functionally impaired people.

## Main text

### Methods

Two studies are included in this report. Study 1 aimed to test the acceptability of magnetic femoral stimulation and reproducibility of TwQ in a small group of older people with sarcopenia; Study 2 aimed to relate magnetic femoral stimulation results to a range of measures of physical function using baseline data from a clinical trial in older people with a history of falls. Both studies followed the principles of the declaration of Helsinki. Study 1 was approved by East of Scotland Research Ethics Committee (approval number 13/ES/0045); Study 2 was approved by Scotland A Research Ethics committee (approval number 13/SS/0086), and was registered as a clinical trial (ISRCTN58995463) with approval from the UK Medicines and Healthcare Regulatory Authority (EudraCT number 2013-001677-24; CTA number 21726/0281/001).

### Study 1

Community dwelling people > 65 years with sarcopenia and self-reported difficulty with one or more activities of daily living (ascertained by an open question to participants) were recruited through the Scottish Primary Care Research Network. We excluded people who had metal implants or metal fragments in the lower limbs, previous internal fixation of lower limb fracture, vascular stents, those with internal cardiac defibrillators, cardiac pacemaker and implanted nerve stimulators; any condition (e.g. severe osteoarthritis) that was likely to be aggravated by lower muscle contraction; cognitive impairment precluding informed consent; inability to mobilise without human assistance and those residing in a nursing home.

Participants attended a baseline and follow up visit after 2 weeks. At the baseline visit, potential participants were screened for sarcopenia, defined as: Total skeletal muscle mass (SMM) measured by bioimpedance [9] of < 10.76 kg/m^2^ for men or < 6.76 kg/m^2^ for women *and* either low handgrip strength (< 30 kg for men or < 20 kg for women) or low gait speed (< 0.8 m/s for the 4 m walk). Body composition was measured using bioimpedance (BIA) (Akern Bodygram Pro 101 v.3.0; Akern Srl, Florence, Italy). The Janssen formula [10] was used to calculate total skeletal muscle mass/height squared (SMM/H^2^). Handgrip strength was measured in the non-dominant hand using a Jamar dynamometer (Lafayette Instruments, Lafayette, Indiana, USA). The best of three attempts was recorded.

### Outcomes

Outcomes were measured at baseline and at 2 weeks. Acceptability of magnetic stimulation was assessed using a visual analog scale, which asked participants to indicate (a) the degree of discomfort whilst undertaking the Magstim test and (b) how tired they felt at the end of the study. Scales were anchored by statements, e.g. 0% was anchored by ‘no discomfort’ and 100% by ‘extreme discomfort’. The final question asked if they would have the magnetic stimulation test again. The time in seconds taken to walk 4 m at their usual pace was assessed, with the faster of two attempts recorded. The Short Physical Performance Battery (SPPB) [11] was performed according to standard protocol. Isometric quadriceps maximum voluntary contraction strength (QMVC) was measured in a semi reclining position with the knee joint at 90 degrees using a Biopac tension dynamometer (Biopac Systems inc. California, USA). The measurement was taken on the right leg unless the participant had a metallic hip joint on this side. An inelastic strap was placed around the ankle and connected to the dynamometer mounted to the couch. The participant was then instructed to kick their foot out, pushing hard against the strap, hold for 10 s and then relax. This procedure was repeated twice. QMVC was taken as the highest mean force that could be sustained over one second. Participants undertook a 6 minute walk test along a 25 m course at their normal walking pace, using a walking aid if needed. Standardised encouragement was given. The distance covered was recorded as a measure of submaximal endurance.

### Magnetic femoral nerve stimulation

The greatest twitch tension generated in the quadriceps (TwQ) was measured using the Magstim 200 system (Magstim Company Ltd., Whitland, UK). A double 70 mm coil in the shape of a figure of eight was used. Participants were placed in a semi reclining position with a strap around the ankle as described before. The crossover point of the coil was then positioned over the groin to stimulate the femoral nerve using a single biphasic stimulus; the femoral artery was palpated to guide placement over the femoral nerve. A range of magnetic outputs (60, 70, 80, 90 and 3 times at 100%) were applied to check supramaximality of TwQ response. A 30 s interval was allowed between each reading.

### Study 2

For study 2, we used baseline data from a double-blind randomised controlled trial of the effect of perindopril, an angiotensin converting enzyme inhibitor (ACEi) on postural stability in older people. Details of the inclusion and exclusion criteria and study population have been reported previously [12]. QMVC was measured as described for Study 1, but fatigability was also assessed by performing repeated QMVC measurements with no break between 3 s efforts. The number of efforts performed before QMVC fell to 70% of maximum was recorded as a measure of endurance. In addition, after initial TwQ testing, three measurements at 100% were performed immediately and after a 10 min rest to estimate the effect of fatigue and recovery on TwQ following repeated QMVCs. Fatigability was further tested by measuring TwQ following the 6 minute walking test described above.

### Statistical analysis

We used Pearson’s correlation coefficients to compare baseline TwQ with other baseline measures of physical function, and used two-way, random effects intraclass correlation coefficients to test reliability, comparing baseline and follow up TwQ. All analyses were performed using SPSS v21 (IBM, New York, USA) and a 2-sided p value of < 0.05 was taken as significant for all analyses.

## Results

### Study 1

26 participants attended screening, of whom 12 (46%) proceeded to the main outcome measures. 14 (54%) had skeletal muscle mass above the threshold for sarcopenia and were not included. Details of the included participants are given in Table 1; all 12 participants successfully underwent magnetic femoral stimulation. After baseline testing, the mean discomfort rating was 9% (range 0–40%) on a visual analog scale. Mean tiredness was 22% (range 0–71%). 11/12 (92%) undergoing magnetic femoral stimulation said they would be happy to undergo the procedure again, with one participant being unsure. At the second test visit, similar results were seen; mean discomfort 6% (range 0–18%) and mean tiredness 21% (range 0–79%).

**Table 1:** Baseline details of study 1 patients (n = 12)

	Baseline value	Correlation with TwQ	
		r	p
Mean age (years) (SD)	77.6 (6.2)	–	–
Male sex (%)	4 (33)	–	–
Mean height-adjusted muscle mass (kg/m^2^) (SD)	7.1 (1.1)	0.53	0.11
Mean 4 m gait speed (m/s) (SD)	0.65 (0.16)	0.74	0.014
Mean six minute walk distance (m) (SD)	272 (116)	0.33	0.35
Mean handgrip strength (kg) (SD)	11.1 (6.2)	0.41	0.24
Mean short physical performance battery (SD)	6.7 (2.3)	0.24	0.51
Mean MVQC (kg) (SD)	12.8 (8.9)	− 0.15	0.68
Mean TwQ (kg) (SD)	1.4 (0.8)	–	–

*MVQC* maximal voluntary quadriceps contraction, *TwQ* quadriceps twitch tension

Table 2 shows the reproducibility of each measure between the first and second visit, using all available data and also excluding those with a clinically significant variation in their six minute walk distance between the first and second visits. Given that some participants clearly had a change in their physical function in the 2 weeks between the first and second visit, we correlated the change TwQ with changes in the other measures of physical performance: change in QMVC r = 0.42 (p = 0.30); change in six minute walk distance r = 0.05 (p = 0.90); change in SPPB r = 0.55 (p = 0.16); change in handgrip r = 0.62 (p = 0.10).

**Table 2:** ICC (2 way, random effects model for absolute agreement) between first and second visit

	ICC (95% CI)	p
6 minute walk distance		
All	0.94 (0.79–0.98)	< 0.001
Stable on 6MW^a^	0.98 (0.92–1.00)	< 0.001
Maximal voluntary quadriceps contraction strength		
All	− 0.27 (− 0.84 to 0.41)	0.77
Stable on 6MW^a^	− 0.63 (− 1.14 to 0.26)	0.92
Maximal quadriceps twitch tension		
All	0.06 (− 0.53 to 0.68)	0.44
Stable on 6MW^a^	− 0.21 (− 0.92 to 0.74)	0.65
Handgrip strength		
All	0.89 (0.65–0.97)	< 0.001
Stable on 6MW^a^	0.85 (0.10–0.97)	< 0.001
Short physical performance battery		
All	0.55 (− 0.08 to 0.86)	0.04
Stable on 6MW^a^	0.62 (− 0.13 to 0.91)	0.05

^a^Excluding those with 6 minute walk (6MW) difference of ≥ 50 m between visits

### Study 2

A total of 80 participants were randomised into the second study; 68/80 (85%) agreed to have magnetic femoral stimulation and analysable data were available from 64/80 (80%). Baseline details have been published previously [12]. Both mean muscle mass (7.4 [SD 1.4]kg/m^2^) and measures of muscle strength (mean MVQC 19.1 [SD 8.5] kg; mean TwQ 2.7 [SD 1.4] kg) were higher in study 2 than in study 1. Table 3 gives correlations between baseline TwQ and measures of physical function, and muscle mass and fat mass in the larger sample from study 2.

**Table 3:** Correlations between baseline maximal Magstim response (TwQ) and measures of muscle function (n = 64)

	Baseline TwQ		TwQ after kicks		TwQ after 6MW	
	r	p	r	p	r	p
QMVC	0.26	0.04	0.23	0.07	0.21	0.11
6 minute walk distance	0.24	0.06	0.19	0.12	0.25	0.05
Height-adjusted muscle mass	0.17	0.20	0.19	0.14	0.17	0.19
Height-adjusted fat mass	− 0.29	0.02	− 0.37	0.003	− 0.31	0.02
Number of kicks to fatigue	− 0.03	0.82	− 0.06	0.64	0.06	0.63

## Discussion

Previous studies in younger populations [13, 14] have found that magnetic femoral nerve stimulation is feasible and acceptable in younger, healthy populations. However, in contrast to our results, Tofari et al. [15] demonstrated good reliability with repeated testing, although testing was done more frequently and at shorter intervals in healthy subjects. Similarly, Vivodtzev et al. [16] demonstrated high reproducibility in repeated testing on the same day in their study of 23 healthy sedentary participants. We noted considerable variability between visits for both TwQ and QMVC, and some variability between visits for the six minute walk distance. It is therefore possible that underlying physical function did in fact change in the 1–2 weeks between visits, making assessment of reliability more difficult. However, the ICC for magnetic femoral nerve stimulation was much worse than for the six minute walk test, SPPB or handgrip strength, suggesting that notwithstanding any real changes in physical function, magnetic femoral stimulation measurements in older people have high intrinsic variability. Conversely, there was only moderate correlation between changes in TwQ and changes in other measures of physical function over time, suggesting that although some differences in TwQ between baseline and follow up may have been due to real changes in function, by no means all of the change in TwQ can be attributed to this. Poor reliability (which translates into a large variance around any change seen in studies) means that large sample sizes are likely to be required to detect meaningful change in clinical studies.

Our results are strengthened by using two different, complementary study populations, one of which comprises older people who have sarcopenia. Previous studies in older patients have looked mainly at healthy populations with a lower mean age than seen in our study. Hamnegard et al. [5] found that the mean TwQ/QMVC ratio in younger men and women (mean age 38 years) was 0.15. This was similar to the ratio we found in our older participants although the mean QMVC and TwQ were both much lower—18.9 versus 70.1 kg and 2.7 versus 9.8 kg respectively. Other studies in healthy volunteers have shown much higher levels of muscle activation relative to maximal voluntary contraction however [17], and inability of femoral magnetic stimulation to fully stimulate skeletal muscle in neuropathic conditions [18] is a potential concern, especially given the contribution that denervation is postulated to make to the pathophysiology of sarcopenia [19].

In conclusion, magnetic femoral nerve stimulation is acceptable and feasible in functionally impaired older people. Our results do not however support using magnetic femoral nerve stimulation for these patients; the variability of measurements, expense and cumbersome nature of the equipment argue against using this technique as a replacement for simple, inexpensive measures of physical function in current use.

## Limitations

Both studies included in this report have limitations. The sample size were small with the main reason for failing screening in study 1 being muscle mass higher than the EWGSOP definition for sarcopenia; this limits our ability to show statistical significance for some correlations but does not change the point estimate for correlations. Participants in study 2 were not selected on the basis of meeting a diagnosis of sarcopenia; the higher mean six minute walk test values suggest that muscle function was not severely impaired in this participant group, and the generalisability of the findings from study 2 are limited by the fact that this population were highly selected by dint of their participation in a clinical trial. We used grip strength in the non-dominant hand in both studies for consistency as this was the measure employed in the clinical trial from which we obtained data, although we acknowledge that dominant side grip, or maximal grip in either hand are more commonly used [20]. A similar limitation applies to our use of the right leg for testing; this was done for consistency and convenience of our equipment set-up, but may have led to underestimation of strength for participants with left leg dominance. All lower limb tests were however performed on the same leg to ensure comparability within an individual.

## Abbreviations

TwQ: Maximum twitch generated in the quadriceps through magnetic stimulation
SMM: Total skeletal muscle mass
SPPB: Short physical performance battery
QMVC: Quadriceps maximum voluntary contraction
ACEi: Angiotensin converting enzyme inhibitor
ICC: Intraclass correlation co-efficient
